# Identification of serum metabolites associated with the EAT-Lancet planetary health diet, and their mediating role in weight status and weight changes in adults

**DOI:** 10.1007/s00394-026-04115-3

**Published:** 2026-10-07

**Authors:** Tiina Suikki, Mirkka Maukonen, Aki S. Havulinna, Rilla Tammi, Anne-Mari Halme, Pirkko Pussinen, Tuulia Kreetta Pietilä, Anne-Maria Pajari, Satu Männistö

**Affiliations:** 1 Finnish Institute for Health and Welfare, Helsinki, Finland; 2 University of Helsinki, Helsinki, Finland; 3 Folkhälsan Research Center, Helsinki, Finland; 4 University of Turku, Turku, Finland; 5 Institute of Dentistry, University of Eastern Finland, Kuopio, Finland

**Keywords:** Diet quality index, Metabolite, Obesity, Planetary health diet, Sustainability, Mediation

## Abstract

**Purpose:**

Better adherence to a healthy and sustainable diet has been associated with a lower body weight, but the underlying metabolic mechanisms remain unclear. We examined whether serum metabolites mediate this association.

**Methods:**

We used data from three Finnish population-based health examination studies (*n* = 9225, 54% of women, mean age of 54 years). Diet quality was assessed with a dietary score based on the Planetary Health Diet (PHD) adapted to Finnish food culture. Weight, height, waist circumference (WC), and body mass index were measured at baseline and follow-up, and their percentual changes over seven years were calculated. Serum metabolites (*n* = 158) were quantified using a high-throughput nuclear magnetic resonance (NMR) spectroscopy metabolomics platform (Nightingale Health Plc). The role of individual metabolites in the diet-anthropometry associations by sex were assessed by mediation analyses using multivariable linear regression. Analyses were replicated in an independent dataset of three additional Finnish studies (*n* = 10242).

**Results:**

Mediation analyses indicated that seven metabolites partly mediated diet-WC association in both men and women: short-chain fatty acid acetate (7.6/3.1%, respectively), degree of fatty acid unsaturation (26.0/23.1%), and several fatty acid-to-total fatty acid ratios (polyunsaturated (35.6/46.5%), docosahexaenoic (5.5/9.6%), omega-3 (4.0/9.8%), linoleic (31.4/49.9%), and saturated fatty acids (12.1/13.0%).

**Conclusion:**

The association between higher PHD adherence and a smaller WC was partly explained by several serum metabolites likely reflecting higher fiber intake and better fat quality of the PHD. These findings suggest that healthy and sustainable diets may contribute to weight management through favourable metabolic profiles.

**Supplementary Information:**

The online version contains supplementary material available at https://doi.org/10.1007/s00394-026-04115-3.

## Introduction

Obesity is a multifactorial disease defined by excessive accumulation of adipose tissue leading to metabolic changes and an increased risk of chronic diseases such as type 2 diabetes, cardiovascular diseases and certain cancers [1]. Therefore, the global increase in average body weight poses a major public health concern [2]. In Finland, for example, in 2023, about 30% of adults were identified as living with obesity [3].

Diet is one of the key modifiable factors influencing body weight. While consumption of individual food groups have been associated with either increased (e.g., red and processed meat) or decreased (e.g., whole grains) risk of obesity [4, 5], whole-diet approaches capture better the complex interplay of foods consumed together. Overall diet quality assessed with different healthy dietary indices has been associated with lower risk of obesity [6, 7]. Recently, research interest has increasingly focused on diets that promote both human health and environmental sustainability. For example, the Planetary Health Diet (PHD), which emphasizes increased consumption of plant-based foods, such as whole grains, vegetables, fruits, legumes, nuts, and seeds, and reduced consumption of animal-based products, including red and processed meats and dairy, has mostly been associated with lower body weight [8–10], although conflicting results have been reported as well [11, 12]. However, in a recent systematic review and meta-analysis of eight observational (cohort and cross-sectional) studies, sustainable diets, such as the PHD and organic diets, were associated with a lower risk of overweight and obesity [13]. In another systematic review of nine prospective cohort studies, plant-based dietary patterns were positively associated with body weight maintenance with moderate level of evidence [7].

Several mechanisms may explain the potential beneficial associations of sustainable diets on body weight. For example, plant-based foods have been found to stimulate energy expenditure through thermogenesis, and to regulate lipid metabolism, gut microbiome composition, and inflammation, which may help reduce obesity risk independently of total energy intake [7]. In the previously mentioned meta-analysis, the results remained consistent even after adjusting for total energy intake and physical activity [13]. Furthermore, higher fiber and polyphenol intake has been found to enhance satiety and weight management [14, 15]. Moreover, individuals who follow plant-based diets tended to have overall healthier lifestyles [13].

Although some potential mechanisms have been identified, the precise molecular pathways linking diets to anthropometric measures remain unclear. Metabolomics provides a powerful tool for examining small-molecule metabolites in biological samples, such as plasma, advancing our understanding of metabolic health and disease pathways [16]. While both overall diets and obesity have been associated with various metabolites [17–20], studies examining the potential mediating role of metabolites, whereby diet is linked to distinct metabolite profiles that may contribute to weight regulation, are scarce. Thus far only one study on Chinese adults (*n* = 4778) have examined and found a partial mediation of several lipid-related circulating metabolites associated with an unhealthy dietary pattern characterized by high intake of rice, red meat, poultry, and fish in the relationship with increased risk of general and central obesity [21]. However, no associations were found between a healthier dietary pattern and plasma metabolites.

In general, evidence on the role of metabolites in mediating the relationship between a priori healthy diets, such as the PHD, and anthropometric measures remains limited. To address this research gap and deepen the understanding of the underlying mechanisms, we aimed to investigate the metabolic pathways linking adherence to the PHD with anthropometric measures through metabolites in the Finnish adult population. Specifically, we first identified metabolites associated with the PHD and then examined their potential mediating role in the relationship between the PHD and weight status and weight changes during follow-up. This study offers a mechanistic perspective that advances current understanding of how plant-based diets can modulate body composition through their effects on metabolic pathways.

## Methods

### Data source

We used data from six Finnish population-based health examination studies (Health 2000, Health 2011, DILGOM 2007, DILGOM 2014, FINRISK 2012, and FinHealth 2017) coordinated by the Finnish Institute for Health and Welfare (THL) (Supplemental Fig. 1). Each of the studies included, for example, collection of blood samples, anthropometric measures, and self-administered questionnaires, such as a food frequency questionnaire (FFQ). In Health 2000, 6771 adults aged ≥ 30 years participated in a health examination and interviews (participation rate 84%) between 2000 and 2001 [22]. Of these, 4006 participated in the follow-up study Health 2011 eleven years later (participation rate 63%) [23]. DILGOM 2007 was conducted between April and June in 2007 as a sub-study of the National FINRISK 2007 Survey, with 5024 participants aged 25–74 years (participation rate 80%). All eligible participants from DILGOM 2007 were invited seven years later to the follow-up study, DILGOM 2014, of which, 3725 took part (participation rate 82%) [24]. In DILGOM 2014, the participants were divided into two groups: 1312 attended a health examination at the study site (group 1), and 2423 conducted an anthropometric self-assessment at home using a mailed measuring tape (group 2) [25]. In FINRISK 2012, 5827 adults aged 25–74 years participated in a health examination and filled in an FFQ in 2012 [26]. FinHealth 2017 was a continuation of the Health 2000/2011 and FINRISK Studies, with 5334 adults aged ≥ 18 years (participation rate 52%) who took part in a health examination and filled in self-administered questionnaires, including the FFQ in 2017 [27]. More detailed descriptions of the study protocols and methodology have been described elsewhere [22–27].

All studies were conducted according to the guidelines of the Declaration of Helsinki. The research protocols were approved by the Ethics Committee of the Hospital District of Helsinki and Uusimaa (Health 2000: 407/E3/2000, Health 2011: 45/13/03/00/11, DILGOM2007: 229/EO/2006, DILGOM 2014: 332/13/03/00/13, FINRISK 2012: 162/13/03/11, FinHealth 2017: 37/13/03/00/2016). All participants signed written informed consent.

### Study population

We included all participants with baseline information on dietary intake (acceptably completed FFQ), weight, and metabolites. Additionally, for the follow-up analyses, we included participants with information on weight at follow-up. In each cohort, participants with implausible energy intake (Health 2000 (*n* = 18): energy intake < 600 or > 7000 kcal/d; FINRISK 2012 (*n* = 48), DILGOM 2007 (*n* = 48), FinHealth 2017 (*n* = 51): 0.5% sex-specific extremes of energy intake distribution) and those pregnant at baseline or follow-up were excluded.

We conducted all analyses in the pooled main sample including participants from FinHealth 2017 and DILGOM 2007/2014 studies, and in the pooled replication sample including participants from FINRISK 2012 and Health 2000/2011 studies. The final main sample comprised 9225 participants (4844 from FinHealth 2017, 4837 from DILGOM 2007, including also 3456 from DILGOM 2014), and the replication sample 10 242 participants (4603 from FINRISK 2012, and 5639 from Health 2000, including also 4138 from Health 2011).

### Dietary data and diet quality

Data on participants’ habitual diet over the last 12 months was collected with a semiquantitative FFQ, which has been repeatedly updated and validated against food records [28–30]. The FFQ was completed either at the study site or at home and mailed to THL. The FFQ included 128–131 food items (depending on dataset) representing commonly consumed foods and beverages in Finland. They were identified from the most recent national food consumption surveys based on dietary interviews (e.g., FinDiet).We used predefined portion sizes (e.g., slice, glass, serving), and participants reported their average consumption frequency using nine categories, ranging from “never or seldom” to “six or more times a day”. Each food item was linked to the Finnish Food Composition Database (Fineli^®^), which is utilized by the in-house software FINESSI to calculate average daily food consumption, as well as energy and nutrient intake [31].

We assessed overall diet quality using the Planetary Health Diet Score (PHDS), which is a diet quality index based on the PHD [8] and adapted for the Finnish food culture [32]. The PHDS includes 13 food group and nutrient components according to which participants are scored: (1) whole grains, (2) all vegetables, (3) potatoes, (4) fruits and berries, (5) liquid dairy and dairy foods as milk equivalents, 6) red and processed meat, (7) poultry, (8) eggs, (9) fish and fish products, (10) legumes, (11) nuts and seeds, (12) ratio of unsaturated and saturated fat intake, and (13) sucrose. Mean daily consumption of these components (except for fat ratio) was energy-standardised by dividing the consumption (g/d) by the participant’s mean total energy intake (kcal/d) and multiplying it by 2500, which is the recommended total daily energy intake (kcal) in the original PHD [8]. Next, each component was assigned either 0 points for not meeting or 1 point for meeting the PHDS pre-defined cut-off values [32] (Supplemental Table 2). Total score could range from 0 to 13 points with higher score indicating better adherence to the PHD. Furthermore, mean consumption with standard deviation of each score component was calculated (without energy-standardization).

### Anthropometric variables

Weight (kg), height (cm), and waist circumference (WC, cm) were measured either by trained study nurses at the study sites, or self-reported (height and weight) or self-measured (WC) by the participants (DILGOM 2014 group 2) according to the international protocols with participants wearing light clothes and no shoes [33]. The precise measuring protocols have been described elsewhere [22, 23, 25, 27, 34]. The self-reported or self-measured anthropometrics have been previously validated against those taken by the study nurses [25]. Body mass index (BMI) was calculated dividing weight (kg) by height squared (m^2^). Percentual changes in weight, BMI, and WC during the follow-up were calculated by dividing the absolute change (follow-up measure – baseline measure) by their baseline measures.

### Metabolomic biomarker profiling

 Serum samples were obtained from whole blood collected and processed at the study sites. The participants were instructed to fast for at least four hours prior to sample collection [22, 27, 34]. The samples were transported to the laboratory for quality check and they were stored at − 70 °C [22, 27, 34]. Serummetabolite profiles were quantified using a high-throughput nuclear magnetic resonance (NMR) spectroscopy metabolomics platform (Nightingale Health Plc, Helsinki, Finland). The profiles provide a snapshot of the individual’s physiological status focusing on lipid metabolism, proteolysis, glycolysis, and ketolysis [35–37]. We focused on 158 biomarkers including lipids, such as triglycerides, phospholipids, free and esterified cholesterol transported by 14 different lipoprotein subclasses, fatty acids, ketone bodies, and amino acids.

### Potential confounding factors

Participants’ age and sex were obtained from the sampling frame (Finnish Population Information System). Education level, leisure-time physical activity, smoking status, and cholesterol medication use were obtained from the self-administered questionnaires. Educational level was harmonized across cohorts into low (elementary school or the lowest tertile of total school years according to sex and birth cohort to adjust for the extension of the basic education system and increase in average school years over the last decades), medium (upper secondary school or the middle tertile of adjusted total school years), and high (university education or the highest tertile of adjusted total school years). Participants were categorised into inactive (light activities such as reading and watching television), moderately active (e.g., walking, cycling, and gardening at least 4 h/wk) and active (e.g., brisk running, swimming, or other physically demanding activities at least 3 h/wk) based on their self-reported leisure-time physical activity. For smoking status, a three-level scale was also used, including current smokers, former smokers, and never smokers. Based on the use of cholesterol-lowering medication, the participants were categorised into users and non-users.

### Statistical analyses

The data were pooled for the analyses, adjusting for study cohort, as we detected no between-cohort heterogeneity based on between-study variance estimated by the DerSimonian-Laird method. Men and women were analysed separately because of different body compositions and different metabolic responses for example to dietary intake [38]. Variables that did not follow a normal distribution were transformed using the cube root. For analyses where the dependent variable was cube-root transformed, the estimates were rescaled with back-transformation . Furthermore, all metabolites were transformed to z-scores (mean 0, SD 1). To handle missing data in covariates (leisure-time physical activity: *n* = 58 in FinHealth 2017 and DILGOM 2007; *n* = 96 in FINRISK 2012 and Health 2000; smoking status: *n* = 69 in FinHealth 2017 and DILGOM 2007; *n* = 55 in FINRISK 2012 and Health 2000), mode imputation was used, replacing missing values with the most frequently occurring value in each variable. Statistical analyses were performed with the R statistical computing program (version 4.09) with using the “meta” package for meta-analysis and “Mediation” package for mediation analyses. To correct for multiple hypothesis testing, the Benjamini-Hochberg procedure for false discovery rate (FDR) was applied in all multiple linear regressions, with an FDR-adjusted *p*-value < 0.05 considered statistically significant.

**Fig. 1:**
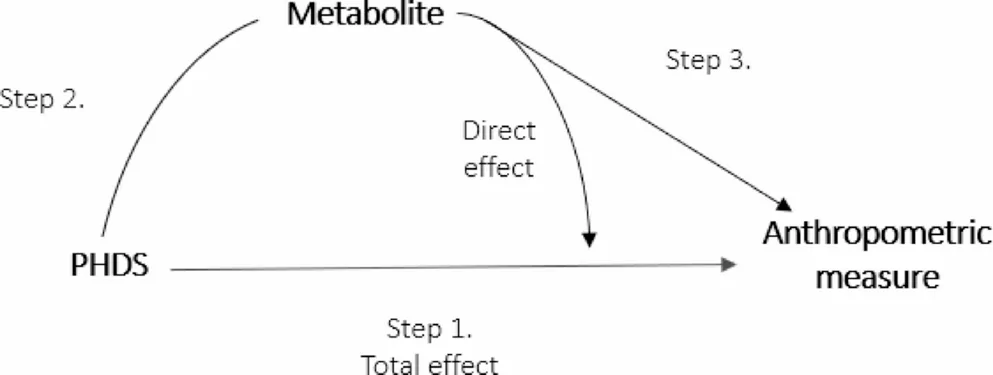
General illustration of mediation analysis following principles by Baron and Kenny (37) based on multiple linear regression analyses. In step 1 was analysed the association between the independent variable (Planetary Health Diet Score, PHDS) and dependent variable (each anthropometric measure at a time) describing the total effect in this association (without consideration of mediator) adjusted with potential covariates. In step 2 was analysed the association between the independent variable (PHDS) and potential mediator (each metabolite at a time) adjusted with potential covariates. In step 3 was analysed the association between the mediator and dependent variable. Direct effect describes the association between the independent variable (PHDS) and dependent variable (anthropometric measure) when the effect of mediator has been taken into account. Mediation effect of mediator was calculated by subtracting the beta estimate of the direct effect from the beta estimate of the total effect

We used multiple linear regression models and mediation with bootstrapping according to the Baron and Kenny mediation test to analyse whether metabolites mediate the association between the PHDS (independent variable) and anthropometric variables (dependent variable) (Fig. 1) [39, 40]. In step 1, linear associations between the PHDS and anthropometric variables (weight, BMI, WC, and their changes during the follow-up) were analysed to establish total effect for the subsequent mediation analysis. In step 2, linear associations between the PHDS and each metabolite were tested. In step 3, linear associations between statistically significant metabolites from step 2 and statistically significant anthropometric variables from step 1 were tested. To detect whether the selected metabolites potentially mediate (direct effect) the associations between the PHDS and the selected anthropometric variables, the linear association in step 1 (total effect) was adjusted for each metabolite at a time. Then the potential mediation effect (average causal mediation effects, ACME) of the selected metabolites was calculated by subtracting the beta estimate of the direct effect from the beta estimate of the total effect. The statistical significance of the mediation effect (FDR-adjusted p-value), and the proportion of mediation were further analysed by using bootstrapping approach with 5000 simulations.

Linear regression analyses were conducted with two main models with potential confounding factors selected based on previous literature, author consensus, and data availability. Model 1 included age and cohort, and model 2 was further adjusted for leisure-time physical activity, smoking status, and the use of cholesterol-lowering medication. As a sensitivity analysis, we further adjusted model 2 for educational level, a commonly used proxy for socioeconomic status in this context [41], given its established association with anthropometric outcomes both in Finland [42] and globally [43]. The mediation analyses were conducted using model 2. Additionally, we analysed linear regression between the statistically significant metabolites and the individual components of the PHDS components, adjusting for model 2, to further explore the associations between the PHDS and metabolite profiles.

## Results

The main sample included 4209 men and 5016 women, with the mean age of 54 years across studies and sexes (Table 1). Approximately one fifth of both men and women were physically inactive in leisure-time. One fifth of men were current smokers, while fewer women were daily smokers. One fifth of men and slightly fewer women used cholesterol-lowering medication. The average PHDS was 3.4 (SD 1.3) for men and 4.1 (SD 1.4) for women. At baseline, the average weight was 85 kg for men and 72 kg for women. The average baseline BMI and WC were 27 kg/m^2^ and 98 cm for men, and 27 kg/m^2^ and 88 cm for women. Mean consumption of each PHDS component by sex is shown in (Table 2).

### Associations between the PHDS and anthropometric measures

In step 1, we observed a statistically significant negative association between the PHDS and the baseline WC in both men and women (Model 2: ß − 0.43, FDR *p*-value 0.002, and ß − 0.50, FDR *p*-value < 0.001, respectively) (Table 3). Additionally, the PHDS was negatively associated with the baseline weight and BMI in women, but not in men (Model 2: weight: ß − 0.041, FDR *p*-value 0.005; BMI: ß − 0.16, FDR *p*-value 0.003) (Table 3). On the contrary, we observed no statistically significant associations between the PHDS and anthropometric changes during seven years of follow-up (FDR *p*-value > 0.05) (Table 3).

### Association between PHDS and serum metabolites

**Fig. 2:**
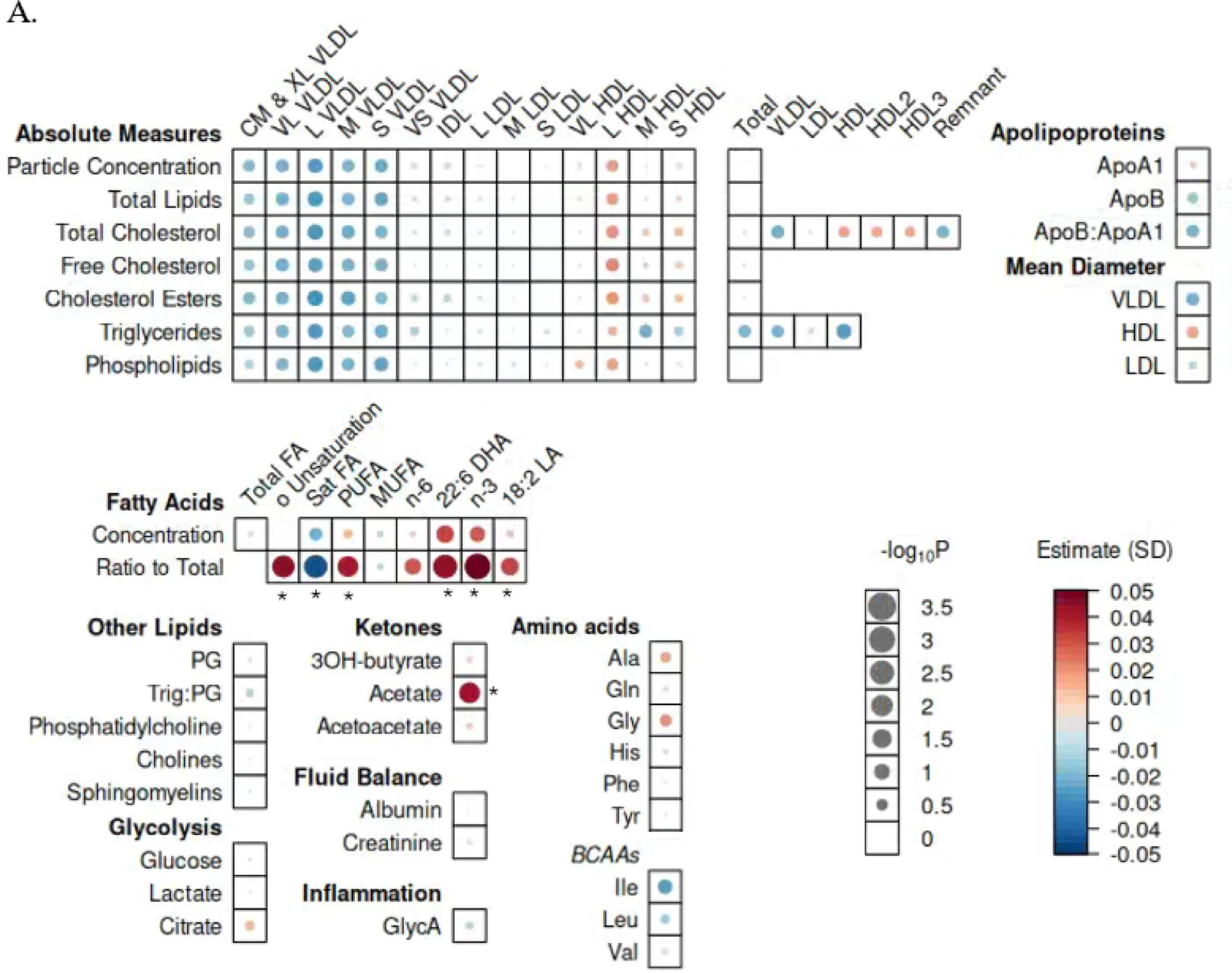

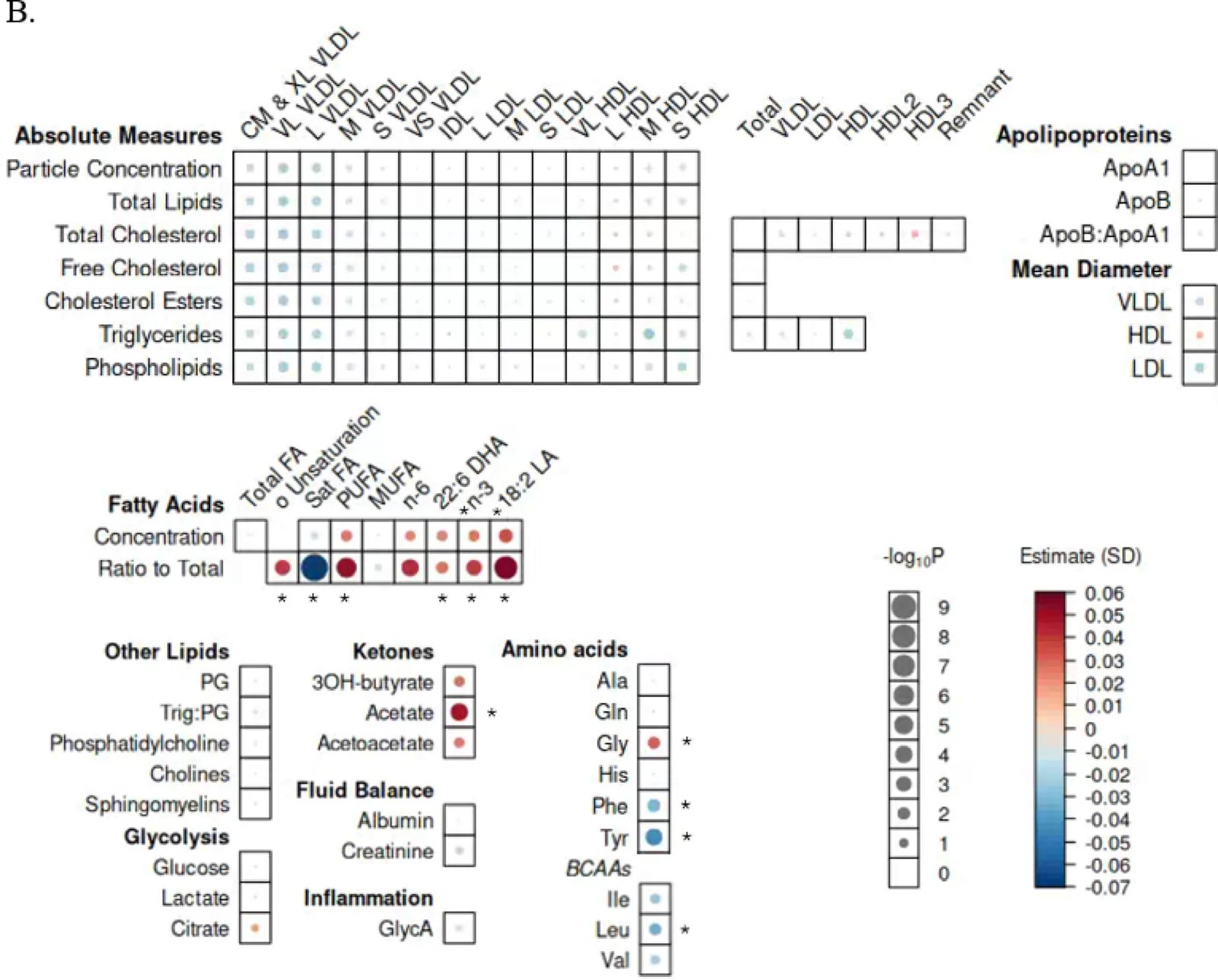
Linear associations between the Planetary Health Diet Score (PHDS) and 158 metabolites transformed to the z-scores (mean 0, SD 1) for men (A) and women (B) in the pooled main dataset of FinHealth 2017 and DILGOM 2007 studies. The association was adjusted with age, cohort, leisure-time physical activity, smoking, and use of cholesterol-lowering medication. Statistically significant false discovery rate (FDR) corrected p-values (< 0.05) were indicated with asterisk (*). Ala, alanine; ApoA1, apolipoprotein A-I; ApoB, apolipoprotein B; BCAA, branched-chain amino acid; DHA, docosahexaenoic acid; FA, fatty acid; Gln, glutamine; Gly, glycine; GlycA, glycoprotein acetyls; HDL, high-density lipoprotein; His, histidine; IDL, intermediate-density lipoprotein; Ile, isoleucine; LA, linoleic acid; LDL, low-density lipoprotein; Leu, leucine; MUFA, monounsaturated fatty acid; Phe, Phenylalanine; PG, phosphoglyceride; PUFA, polyunsaturated fatty acid; SFA, saturated fatty acid; Trig, triglycerides; Tyr, tyrosine; Val, valine; VLDL, very-low-density lipoprotein

In step 2, the PHDS had a statistically significant positive association with six metabolites in men and women: acetate, degree of fatty acid unsaturation, the ratio of 22:6 docosahexaenoic acid to total fatty acids (DHA/FA), the ratio of 18:2 linoleic acid to total fatty acids (LA/FA), the ratio of omega-3 fatty acids to total fatty acids (omega-3/FA), and the ratio of polyunsaturated fatty acids to total fatty acids (PUFA/FA) (Fig. 2A and B). A statistically significant negative association was observed with the ratio of saturated fatty acids to total fatty acids (SFA/FA) in men and women (Fig. 2A and B). Additionally in women, the PHDS was positively associated with the concentrations of LA, omega-3 FA, and glycine, and negatively with leucine, phenylalanine, and tyrosine (Fig. 2B). Furthermore, all aforementioned metabolites were either positively or negatively associated with several components of the PHDS (Supplemental Fig. 3).

### Associations between serum metabolites and anthropometric measures

In step 3, all these metabolites were either positively (phenylalanine, leucine, tyrosine, omega-3 FA, SFA/FA, LA) or negatively (omega-3 FA/FA, DHA/FA, acetate, glycine, degree of fatty acid unsaturation, PUFA/FA, LA/FA) associated with WC, weight, and BMI, except for the concentration of LA regarding weight and BMI (Supplemental Fig. 2).

### Mediation analyses

Since we observed statistically significant association in both men and women only between the PHDS and WC (step 1), we omitted other anthropometric variables from the mediation analyses. Additionally in women, statistically significant associations were observed for weight and BMI.

In the mediation analyses, we adjusted the association between the PHDS and WC for each metabolite found statistically significant in step 2. Following the adjustments, the associations between the PHDS and WC were attenuated but did not completely disappear, indicating partial mediation effect in both men and women, except for LA and omega-3 FA in women (Table 4). Additionally, the results were similar for weight and BMI in women indicating partial mediation for most of the selected metabolites (Supplemental Table 1). All these partial mediation effects were statistically significant, except for those of omega-3/FA in the association between the PHDS and WC in men and of LA in the association between PHDS and BMI in women. For the statistically significant mediation effects, the proportion of mediation varied between 5.5% and 35.6% in men and − 3.7% and 49.9% in women (Table 4, Supplemental Table 1).

### Results from the replication data

The replication data included 4674 men and 5568 women, with a mean age of 52 years across studies and sexes (Supplemental Table 2). The participant characteristics were similar in the replication data to those in the main dataset, although the average PHDS points were slightly lower for both men and women. The mean consumption of each score component is shown in the supplementary material (Supplemental Table 3).

In step 1, we observed statistically significant association between the PHDS and WC for women only (Model 2: ß -0.40, FDR p-value 0.01) (Supplemental Table 4). In step 2, the PHDS was positively associated with two metabolites (acetate and omega-3 FA/FA), and negatively with one metabolite (SFA/FA) (Supplemental Fig. 4). In step 3, acetate was negatively, while omega-3/FA and SFA/FA were positively associated with WC in women (Supplemental Fig. 5). Furthermore, these metabolites were either positively or negatively associated with several components of the PHDS (Supplemental Fig. 6). Similarly to the main data, after adjusting the association between the PHDS and WC (step 1) for each metabolite at a time, the associations were attenuated but did not completely disappear, indicating partial medication. The partial mediations were found statistically significant with the mediated proportions ranging from 5.1 to 8.2% (Supplemental Table 5).

## Discussion

In this study, we identified seven serum metabolites related to energy production and fatty acid metabolism (acetate, degree of fatty acid unsaturation, DHA/FA, LA/FA, omega-3 FA/FA, PUFA/FA, and SFA/FA) that partially mediated the negative associations between the PHDS and WC both in Finnish men and women. Additionally, we identified six metabolites related to polyunsaturated FAs and amino acid metabolism (LA, omega-3 FA, phenylalanine, tyrosine, glycine, and leucine) that mediated this association in women. Similar results were found in the replication dataset, although only for three metabolites and only in women. This may be attributable to the lower mean PHDS points in the replication dataset (3.3 (SD 1.2) for men and 3.7 (SD 1.3) for women) compared to the main dataset (3.4 (SD 1.3) for men and 4.1 (SD1.4) for women).

The potential mediating role of blood metabolites has not been previously studied in the associations between overall diet quality, assessed using a priori dietary scores, and anthropometric measures. However, one previous study of 4778 Chinese adults studied the associations between data-driven dietary patterns, anthropometric measures and circulating lipid-related metabolites analysed with NMR, and metabolites’ mediating role in the association between diet and anthropometrics [21]. In that study, a “balanced” dietary pattern, characterized by the consumption of red meat, poultry, fish, fresh fruits and vegetables, dairy, eggs, and soybean, was not statistically significantly associated with the metabolites. However, a positive linear association was detected between an unhealthy-considered “southern type” dietary pattern characterized by high intake of rice, red meat, poultry, and fish, and 49 metabolites, including different very low-density lipoprotein (VLDL) particle sizes and total lipids in these particles, apolipoproteins, and different fatty acids. Furthermore, these metabolites were associated with a higher risk of obesity [21]. They also found that the analysed metabolites (*n* = 129) explained 30 to 53% of the association between the unhealthy dietary pattern and the risk of general and central obesity [21].

The observed differences between the current study and the Chinese study in the associations between healthier diets and metabolites are likely attributable to the distinct contents of the diets related to dietary assessment methods; while we employed an a priori dietary index based on predefined guidelines (the PHD), the other utilized a data-driven dietary pattern approach. However, a priori healthy dietary patterns have been linked with different NMR-based metabolites [44, 45]. For example, in a study conducted in two datasets from the United Kingdom (*n* = 4824, mean age of 55.9 years) and Finland (*n* = 1716, mean age of 37.7 years), positive associations were observed between the Alternative Healthy Eating Index (AHEI) score and 40 metabolites mainly involved in fatty acid metabolism, such as higher fatty acid unsaturation degree and the higher proportions of polyunsaturated FA, omega-3 and DHA metabolites [44]. These findings were similar to ours, although we observed fewer significant associations between the PHDS and metabolites (*n* = 13). This may be partly explained by the generally low adherence to the PHD and the narrow range of PHDS score in our datasets, which may have attenuated the observed associations with the metabolites, although some associations were nevertheless identified. The low overall PHDS score can result from different combinations of components across individuals, which may introduce variability and obscure associations with the metabolites.

In our study, higher adherence to the PHDS was positively associated with higher serum concentrations of acetate, a small metabolite with diverse roles in both cellular and systemic metabolism including energy, lipid, and protein metabolism [46]. In addition to the hydrolysis of acetyl-CoA and breakdown of acetylated molecules, acetate is primarily produced by microbial fermentation of dietary fiber [47]. Acetate likely reflects increased soluble fiber intake, given that the PHDS emphasizes the consumption of whole grains, fruits and berries, vegetables, and legumes [8]. Furthermore, acetate partially mediated the negative association between the PHDS and anthropometric measures, particularly WC. Previous evidence from both human randomized clinical trials (RCT) and animal studies supports our findings, showing that a higher concentration of acetate is associated with a lower body weight, potentially due to acetate’s regulatory role in energy metabolism, insulin sensitivity, glucose homeostasis, and appetite regulation and satiety [46, 48]. However, emerging evidence also suggests that acetate may promote weight gain under certain conditions, especially in individuals with overweight or obesity [49]. Nevertheless, we found that higher acetate concentrations were associated with lower anthropometric measures.

In our study, the higher adherence to the PHDS was positively associated with the metabolites reflecting greater FA polyunsaturation (unsaturation degree, the ratio of overall PUFAs, omega-3 FAs, DHA, LA to total FAs), and negatively with the saturated FAs (SFA/total FA ratio). These associations were more evident for the FA ratios than for the absolute concentrations, which may better capture dietary fat quality. However, absolute FA concentrations are considered more suitable for assessing changes in fat intake [50]. These findings likely reflect the PHD’s emphasis on reducing animal-based foods – particularly red and processed meat and dairy – and increasing plant-based fat sources [8]. Notably, these FA measures mediated the negative association between the PHDS and anthropometric measures. Accordingly, in a previous longitudinal study a higher intake of PUFAs was linked with a lower weight gain, whereas SFAs were linked with a higher weight gain [51]. This may be explained, for example, by SFAs role in increasing the rate of lipolysis and insulin resistance [49]. In contrast, high intake of PUFAs, especially omega-3 FAs, has been associated with an improved insulin sensitivity and beneficial epigenetic modifications [52–54]. Furthermore, previous RCTs and cross-sectional studies have found that especially fat quality was associated with fat distribution in the body [52, 55, 56]. For example, in a cross-sectional population-based study of British adults (*n* = 9119, 40–69 year old adults from UK Biobank), substituting SFAs with PUFAs and animal-based fats with plant-based fats was beneficial for the fat distribution in the body, as higher intake of SFAs was associated with metabolically more harmful liver fat [56].

Furthermore, we found that in women, higher adherence to the PHDS was negatively associated with the concentrations of three amino acids (AAs): leucine (branched-chain amino acid [BCAA]), phenylalanine, and tyrosine, and positively with the concentration of glycine. Leucine and phenylalanine are essential AAs, while tyrosine (for which phenylalanine serves as a precursor) and glycine are nonessential AAs [57]. The associations between the PHDS and lower AA concentrations are likely explained by the PHD’s emphasis on reducing the consumption of animal-based foods, which are major sources of protein and essential amino acids [58]. This may be more pronounced in women, as in Finland, women tend to consume less animal-based foods than men [59]. In this study, the PHDS was positively associated with glycine concentration, potentially reflecting microbiome-mediated availability and sources linked to plant-based diets [60]. Higher glycine levels also mediated the negative association between the PHDS and anthropometric measures and were independently associated with lower anthropometrics. These findings align with previous evidence linking glycine to improved metabolic health, especially insulin sensitivity [61, 62]. The negative associations between the PHDS and other AAs, as well as their mediating role in the associations between the PHDS and anthropometrics, show some inconsistency with previous literature. While high-protein diets and higher intake of specific AAs have been associated with healthier weight status in some studies [63, 64], other studies have reported associations between lower AA levels and a lower body weight [65, 66]. In our study, higher levels of leucine, phenylalanine, and tyrosine were associated with higher weight, BMI, and WC analysed independently of the PHDS. These findings are consistent with earlier studies linking these AAs to increased WC and BMI [67]. Nevertheless, further studies are warranted to study causality of these associations.

### Strengths and limitations

The main strengths of this study included the large number of participants drawn from several population-based cross-sectional and follow-up studies as well as the replication of the main analyses in an independent dataset. We also conducted the analyses using several outcome variables. Furthermore, the analyses were done by sex, which is important considering that metabolite profiles, body fat distribution, dietary assessment in the questionnaires and diet have been found to diverge between sexes [38, 68]. Also, dietary assessment using a validated FFQ is a strength, although we acknowledge its possible limitations including potential reporting and memory bias. Recognizing the importance of energy intake in relation to weight status and weight change, we standardized the PHDS components to a 2500 kcal intake for all participants, as recommended in the original PHD [8]. Targeted serum metabolomic analyses were conducted with standardized methods by Nightingale [36, 37], which adds to the methodological robustness. Although targeted analytical methods provide limited metabolite coverage, they offer greater sensitivity and accuracy compared to untargeted approaches. In particular, NMR-based analysis of serum fatty acids has been shown to serve as a reliable biomarker for the consumption of dietary fats. This platform, however, has a limited ability to detect metabolites related to some foods, such as fruits and vegetables, or ones in low quantity, highlighting the value of complementary analytical techniques when assessing overall diet quality [16, 44, 50].

We acknowledge potential limitations as well. The possibility of selection bias cannot be excluded as health-conscious people tend to participate more often in health examination studies. Furthermore, the mediating effects of certain metabolites on the associations between the PHDS and anthropometric measures may overlap, as many of the significant metabolites were related to PUFAs. Despite possible correlations among these metabolites, we chose to analyse the mediating role of each metabolite separately to capture their distinct mechanistic contributions. As the mediation analyses were based on cross-sectional data, causality cannot be established, and the mediating role of the identified metabolites should be considered suggestive rather than definitive. Furthermore, as the present study focused on food consumption rather than nutrient intake when assessing adherence to the PHD, the potential contributions of other relevant dietary components, such as micronutrients, polyphenols, and other dietary bioactive compounds, were not evaluated. While some studies have suggested that higher adherence to the PHD may be associated with inadequate intakes of certain micronutrients, such as vitamin B12, calcium, iron, and zinc, the evidence remained inconclusive [69–72]. Consequently, their potential contributions to the observed associations cannot be ruled out and warrants further investigation. Although multivariable models were applied, residual confounding cannot be fully excluded. Body weight, particularly central obesity, may influence the metabolome, thereby complicating the interpretation of associations between diet and metabolite concentrations. For instance, elevated acetate or amino acids concentrations may reflect higher body weight rather than dietary factors. However, adjusting for body weight in subsequent mediation analyses could have led to over-adjustment, potentially masking true mediating effects in further steps. To better understand the role of body weight in shaping the metabolome, future studies could stratify participants by anthropometric measures. Additionally, integrating microbiome and metabolomic data from multiple biological fluids (e.g., faeces and urine) may offer more comprehensive insights into these associations [73, 74] Moreover, information on medications other than cholesterol-lowering drugs was not available, which may have resulted in residual confounding. Nevertheless, the analyses were adjusted for cholesterol-lowering medication use because of its direct influence on the metabolisms of lipids and lipoproteins, a major component of the metabolomics platform used in this study. The use of self-measured anthropometric data in the follow-up study (DILGOM 2014) may have contributed to attenuation of the observed associations. Lastly, our results are limited to the Finnish population, although, the findings may be cautiously generalizable to populations with similar genetic backgrounds, dietary patterns and lifestyle characteristics typical of Nordic and other Western populations.

**Table 1:** Baseline and follow-up characteristics of all participants and according to the studies included in the main dataset of FinHealth 2017 and DILGOM 2007 and 2014 studies

	All		FinHealth 2017		DILGOM 2007	
	Men	Women	Men	Women	Men	Women
Baseline						
Number of participants, n (%)	4209 (46)	5016 (54)	2021 (46)	2823 (54)	2262 (47)	2575 (53)
Age, years	54 (14)	54 (15)	56 (16)	56 (16)	53 (13)	52 (14)
Leisure-time physical activity^a^						
Inactive, %	20	22	21	25	19	19
Moderate, %	49	52	46	49	52	55
Active, %	31	26	33	26	29	26
Smoking status						
Current smoker, %	19	14	17	13	21	14
Former smoker, %	34	20	33	21	34	20
Non-smoker, %	47	66	50	66	45	66
Cholesterol medication						
Users, %	20	14	20	15	19	13
Dietary information						
Daily energy intake, KJ	10,751 (3832)	8677 (3028)	9724 (3251)	7908 (2626)	11,699 (4076)	9447 (3203)
Planetary health diet score^b^	3.4 (1.3)	4.1 (1.4)	3.4 (1.3)	4.1 (1.5)	3.5 (1.2)	4.1 (1.3)
Anthropometric information						
Height, cm	176 (7)	163 (6)	177 (7)	163 (7)	176 (7)	163 (6)
Weight, kg	85 (15)	72 (15)	87 (15)	72 (15)	84 (14)	71 (14)
BMI, kg/m^2^	27 (4)	27 (5)	28 (4)	27 (5)	27 (4)	27 (5)
WC, cm	98 (12)	88 (14)	99 (13)	89 (14)	97 (12)	87 (13)
Follow-up					DILGOM 2014	
Weight change, %^c^					0.9 (6.9)	1.6 (8.0)
BMI change, %^d^					0.3 (6.9)	1.0 (8.1)
WC change, %^e^					2.1 (6.0)	1.8 (6.9)

KJ, kilojoule; BMI, body mass index; WC, waist circumference

Data is presented as means with standard deviations (SD) for continuous variables or percentages for categorical variables

^a^Self-reported leisure-time physical activity: inactive (light activities such as reading), moderately active (walking/cycling at least 4 h/wk), active (physically demanding activities at least 3 h/wk)

^b^Score could range from 0 to 13 points

^c^n_men_ = 1582, n_women_ = 1874

^d^n_men_ = 1582, n_women_ = 1874

^e^n_men_ = 538, n_women_ = 702

**Table 2:** Baseline consumption of the planetary health diet score components in the pooled main dataset of FinHealth 2017 and DILGOM 2007 studies

	Mean consumption (SD)		Scoring the PHDS
	Men	Women	
Baseline			
Planetary health diet score	3.4 (1.3)	4.1 (1.4)	Maximun 13 p
Wholegrains^a^, g/d	75 (50)	68 (44)	1 *p* ≥ 232 g/d
			0 *p* < 232 g/d
Vegetables, g/d	256 (176)	317 (210)	1 *p* ≥ 300 g/d
			0 *p* < 300 g/d
Potatoes, g/d	157 (112)	101 (74)	1 *p* ≤ 100 g/d
			0 *p* > 100 g/d
Fruit and berries, g/d	193 (186)	251 (208)	1 *p* ≥ 200 g/d
			0 *p* < 200 g/d
Dairy and dairy foods^b^, g/d	933 (498)	822 (433)	1 *p* ≤ 500 g/d
			0 *p* > 500 g/d
Red and processed meat, g/d	162 (104)	99 (69)	1 *p* ≤ 28 g/d
			0 *p* > 28 g/d
Poultry, g/d	39 (41)	38 (42)	1 *p* ≤ 58 g/d
			0 *p* > 58 g/d
Eggs, g/d	35 (34)	28 (26)	1 *p* ≤ 25 g/d
			0 *p* > 25 g/d
Fish, g/d	60 (55)	45 (45)	1 *p* ≤ 100 g/d
			0 *p* > 100 g/d
Legumes, g/d	15 (16)	15 (22)	1 *p* ≥ 75 g/d
			0 *p* < 75 g/d
Nuts and seeds, g/d	4 (8)	5 (10)	1 *p* ≥ 25 g/d
			0 *p* < 25 g/d
Sucrose, g/d	53 (34)	47 (28)	1 *p* ≤ 31 g/d
			0 *p* > 31 g/d
Ratio PUFA to SFA	1.4 (0.3)	1.4 (0.4)	1 *p* ≥ 2.0
			0 *p* < 2.0

PHDS, planetary health diet score; PUFA, polyunsaturated fatty acids; SFA, saturated fatty acids

^a^Rye, oats, and barley were chosen to reflect whole grains in the Finnish diet

^b^Dairy and dairy products included liquid milk and other dairy products (e.g., cheese and cream) as milk equivalents: for cream and ice cream equivalent was 2.7, for cheese products 5, and for butter 6.5 according to the equivalents used in the Stockholm Resilience Centre Nordic report [7]

**Table 3:** Linear association between the Planetary Health Diet Score (PHDS) and anthropometrics by sex in the pooled main dataset of FinHealth 2017 and DILGOM 2007 and 2014 studies

		Men					Women				
		ß	95% CI		*p* ^a^	FDR^b^	ß	95% CI		*p* ^a^	FDR^b^
			Lower	Upper				Lower	Upper		
Weight	Model 1	− 0.30	− 0.60	0.01	0.06	0.07	− 0.60	− 0.86	− 0.33	< 0.001	**< 0.001**
	Model 2	− 0.28	− 0.58	0.01	0.06	0.07	− 0.41	− 0.67	− 0.15	0.002	**0.005**
BMI	Model 1	− 0.09	− 0.19	0.01	0.07	0.08	− 0.23	− 0.33	− 0.13	< 0.001	**< 0.001**
	Model 2	− 0.09	− 0.18	0.01	0.07	0.08	− 0.16	− 0.26	− 0.06	0.001	**0.003**
WC	Model 1	− 0.50	− 0.78	− 0.22	< 0.001	**0.001**	− 0.71	− 0.97	− 0.46	< 0.001	**< 0.001**
	Model 2	− 0.43	− 0.70	− 0.17	0.001	**0.002**	− 0.50	− 0.74	− 0.25	< 0.001	**< 0.001**
Weight change, %	Model 1	0.00	− 0.27	0.27	0.99	0.99	− 0.03	− 0.30	0.24	0.83	0.99
	Model 2	− 0.01	− 0.28	0.26	0.93	0.99	0.01	− 0.26	0.28	0.95	0.99
BMI change, %	Model 1	0.02	− 0.25	0.29	0.90	1.00	− 0.04	− 0.31	0.24	0.80	1.00
	Model 2	0.01	− 0.26	0.28	0.96	1.00	0.01	− 0.26	0.28	0.93	1.00
WC change, %	Model 1	− 0.26	− 0.68	0.16	0.22	0.45	− 0.02	− 0.39	0.36	0.93	0.98
	Model 2	− 0.29	− 0.71	0.14	0.19	0.45	− 0.01	− 0.38	0.37	0.98	0.98

BMI, body mass index; FDR, false discovery rate; WC, waist circumference

^a^P-value for linear association

^b^FDR by Benjamini-Hochberg procedure, FDR-value < 0.05 was considered statistically significant (significant results bolded)

Model 1 adjusted with age and cohort, and further anthropometric change with baseline measure (weight/BMI/WC) depending on which of these was examined. Weight change was additionally adjusted for baseline height

Model 2 is Model 1 further adjusted for leisure-time physical activity, smoking, and use of cholesterol-lowering medication

**Table 4:** Mediating role of selected metabolites in the association between the Planetary Health Diet Score (PHDS) and waist circumference (WC) in men and women in the pooled main dataset of FinHealth 2017 and DILGOM 2007 studies

Metabolite	Total effect^a^ (WC ~ PHDS)		Direct effect^b^ WC ~ PHDS + metabolite		Mediation effect^c^	Proportion mediated, %^d^	FDR
	ß (SE)	FDR	ß (SE)	FDR			
Men							
Acetate	− 0.43 (0.002)	0.002	− 0.40 (0.002)	0.004	− 0.03	7.6	**< 0.001**
Estimated degree of unsaturation			− 0.32 (0.002)	0.017	− 0.11	26.0	**0.001**
DHA/FA			− 0.41 (0.002)	0.004	− 0.02	5.5	**0.003**
LA/FA			− 0.30 (0.002)	0.021	− 0.13	31.4	**0.007**
Omega-3/FA			− 0.42 (0.002)	0.003	− 0.01	4.0	0.08
PUFA/FA			− 0.28 (0.002)	0.031	− 0.15	35.6	**0.003**
SFA/FA			− 0.38 (0.002)	0.006	− 0.05	12.1	**0.002**
Women							
Acetate	− 0.50 (0.002)	< 0.001	− 0.48 (0.002)	0.001	− 0.02	3.6	**0.040**
Estimated degree of unsaturation			− 0.38 (0.002)	0.003	− 0.12	23.1	**< 0.001**
DHA/FA			− 0.45 (0.002)	0.001	− 0.05	9.6	**0.002**
18:2, linoleic acid (LA)			− 0.51 (0.002)	< 0.001	0.01	− 3.7	**0.006**
LA/FA			− 0.25 (0.002)	0.039	− 0.25	49.9	**< 0.001**
Omega-3			− 0.52 (0.002)	< 0.001	0.02	− 4.2	**0.007**
Omega-3/FA			− 0.45 (0.002)	0.001	− 0.05	9.8	**< 0.001**
PUFA/FA			− 0.26 (0.002)	0.028	− 0.24	46.7	**< 0.001**
SFA/FA			− 0.43 (0.002)	0.002	− 0.07	13.0	**< 0.001**
Phenylalanine			− 0.37 (0.002)	0.003	− 0.13	25.9	**0.003**
Tyrosine			− 0.40 (0.002)	0.002	− 0.10	18.4	**< 0.001**
Glycine			− 0.45 (0.002)	0.001	− 0.05	8.8	**0.006**
Leucine			− 0.38 (0.002)	0.003	− 0.12	24.1	**0.004**

DHA, docosahexaenoic acid; FA, fatty acid; FDR, false discovery rate; LA, linoleic acid; PUFA, polyunsaturated fatty acid; SFA, saturated fatty acid

Results are presented as beta coefficients with standard errors (SE), and FDR corrected p-values with significance level 0.05 (significant results bolded)

All associations adjusted with age, cohort, leisure-time physical activity, smoking, and use of cholesterol-lowering medication

^a^Total effect indicating the association between the PHDS and WC without the mediator (metabolite)

^b^Direct effect indicating the association between the PHDS and WC adjusted additionally with the mediator (each metabolite at a time)

^c^Mediation effect indicating the mediating role of the mediator

^d^Proportion mediated indicating the proportion that mediator explained the association between the PHDS and WC

## Conclusions

We observed a potential mediating role of acetate, degree of fatty acid unsaturation and the proportions of different PUFAs and SFAs on the negative associations between the PHDS and anthropometrics, especially for WC, in Finnish men and women. Specifically, higher concentrations of acetate and greater fatty acid unsaturation may represent favourable metabolic pathways linking diet quality to central adiposity, suggesting that healthy and sustainable diets may contribute to both weight management and metabolic functions. However, given the exploratory nature of this study, further research in diverse populations is warranted to confirm these findings and assess their generalizability.

## Supplementary Information

Below is the link to the electronic supplementary material.

Supplementary Material 1

## Data Availability

Data described in the manuscript, code book, and analytic code will be made available upon request pending application and approval through a written application to the Finnish Institute for Health and Welfare Biobank (https://thl.fi/en/web/thl-biobank).
