# Supplementary material for: Identification of serum metabolites associated with the EAT-Lancet planetary health diet, and their mediating role in weight status and weight changes in adults
Source: Eur J Nutr. 2026 Oct 7;65(8):269. doi: 10.1007/s00394-026-04115-3 (PMC13645858; doi:10.1007/s00394-026-04115-3)
Supplement: Supplementary file 1 [file 394_2026_4115_MOESM1_ESM.pdf]

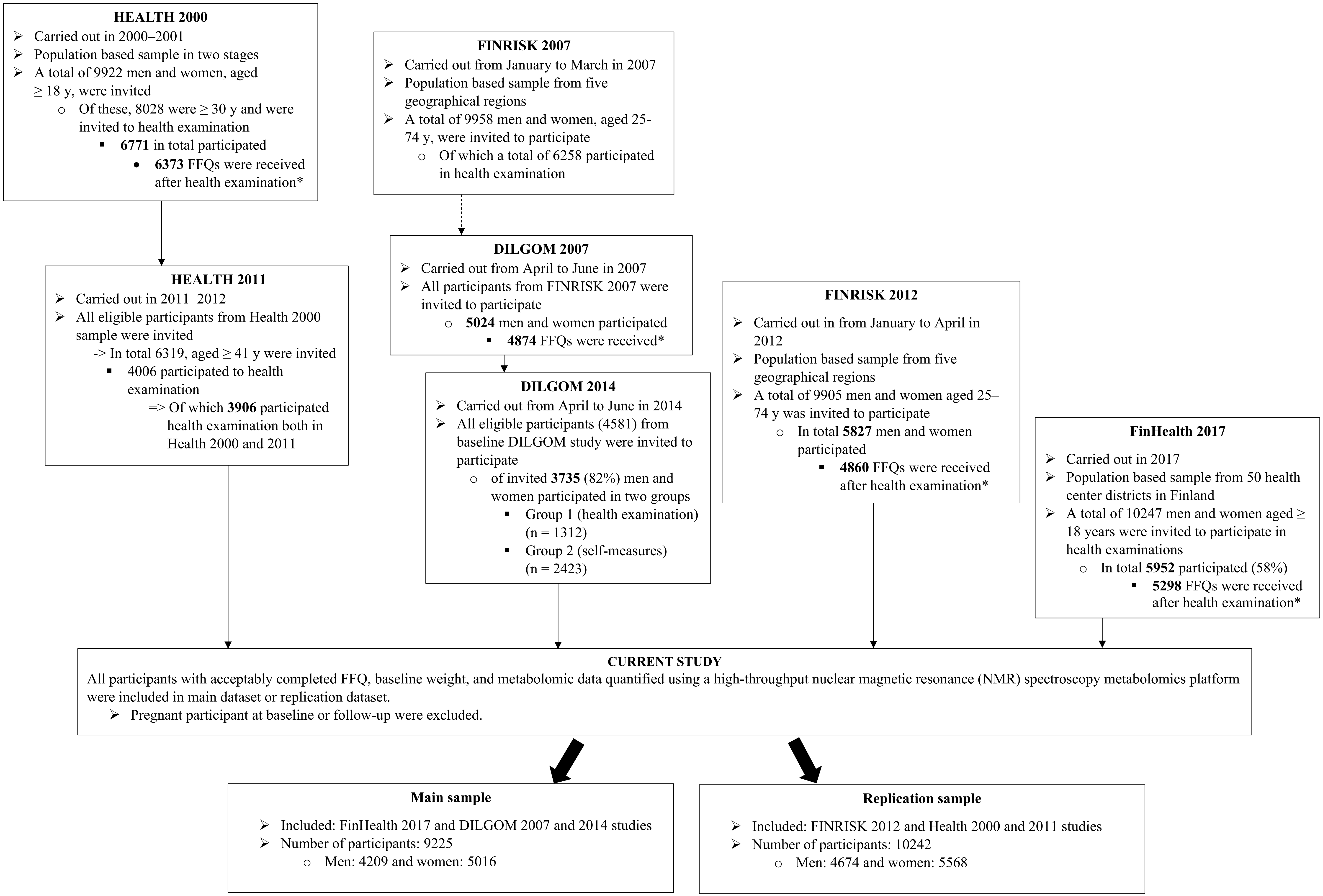

Supplemental figure 1. Flowchart of the included studies (Health 2000 [1] and 2011 [2], DILGOM 2007 [3] and 2014 [4], FINRISK 2012 [5], and FinHealth 2017 [6]).
*Incompletely filled FFQs, duplicate answers, withdrawal of the written consent to participate, and participants with implausible energy intake (Health 2000: energy intake < 600 or > 7000 kcal/d; FINRISK 2012, DILGOM 2007, FinHealth 2017: 0.5% sex-specific extremes of energy intake distribution) were excluded.

Supplemental table 1. Mediating role of selected metabolites in the association between the Planetary Health Diet Score (PHDS) and baseline weight and body mass index (BMI) for women in the pooled main dataset of FinHealth 2017 and DILGOM 2007 studies.

	Total effect[1] (anthropometrics ~ PHDS)		Direct effect[2] (anthropometrics ~ PHDS + metabolite)				
Metabolite	ß (SE)	FDR	ß (SE)	FDR	Mediation effect[3]	Proportion mediated, %[4]	FDR
**Weight**							
Acetate	-0.413 (0.003)	0.005	-0.385 (0.003)	0.05	-0.028	6.8	**0.005**
Estimated degree of unsaturation			-0.308 (0.003)	0.05	-0.105	25.4	**< 0.001**
DHA/FA			-0.368 (0.003)	0.02	-0.045	10.8	**0.003**
18:2, linoleic acid (LA)			-0.427 (0.003)	0.006	0.014	-3.4	**0.040**
LA/FA			-0.172 (0.002)	0.21	-0.241	58.5	**< 0.001**
Omega-3			-0.431 (0.003)	0.005	0.018	-4.4	**0.006**
Omega-3/FA			-0.366 (0.003)	0.02	-0.047	11.3	**< 0.001**
PUFA/FA			-0.194 (0.002)	0.16	-0.219	53.1	**< 0.001**
SFA/FA			-0.340 (0.003)	0.03	-0.073	17.7	**< 0.001**
Phenylalanine			-0.281 (0.002)	0.06	-0.132	32.0	**0.003**
Tyrosine			-0.317 (0.003)	0.05	-0.096	23.2	**< 0.001**
Glycine			-0.376 (0.003)	0.02	-0.037	9.1	**0.006**
Leucine			-0.298 (0.003)	0.05	-0.115	27.8	**0.001**
**BMI**							
Acetate	-0.16 (0.002)	0.003	-0.15 (0.002)	0.01	-0.01	6.7	**0.004**
Estimated degree of unsaturation			-0.12 (0.002)	0.04	-0.04	24.7	**< 0.001**
DHA/FA			-0.14 (0.002)	0.01	-0.02	10.5	**0.004**
18:2, linoleic acid (LA)			-0.17 (0.002)	0.004	0.01	-3.2	0.05
LA/FA			-0.07 (0.002)	0.19	-0.09	57.4	**< 0.001**
Omega-3			-0.17 (0.002)	0.004	0.01	-4.2	**0.007**
Omega-3/FA			-0.14 (0.002)	0.02	-0.02	11.1	**0.002**
PUFA/FA			-0.08 (0.002)	0.15	-0.08	52.1	**< 0.001**
SFA/FA			-0.13 (0.002)	0.03	-0.03	17.3	**< 0.001**
Phenylalanine			-0.11 (0.002)	0.05	-0.05	31.4	**0.003**
Tyrosine			-0.12 (0.002)	0.04	-0.04	22.5	**< 0.001**
Glycine			-0.15 (0.002)	0.01	-0.01	8.9	**0.003**
Leucine			-0.12 (0.002)	0.04	-0.04	26.7	**0.002**

DHA, docosahexaenoic acid; FA, fatty acid; FDR, false discovery rate; LA, linoleic acid; PUFA, polyunsaturated fatty acid; SFA, saturated fatty acid.
Results are presented as beta coefficients with standard errors (SE), and FDR corrected p-values with significance level of 0.05 (significant results bolded).
All associations adjusted with age, cohort, leisure-time physical activity, smoking, and use of cholesterol-lowering medication.
[1]Total effect indicating the association between the PHDS and weight or BMI without the mediator (metabolite).
[2]Direct effect indicating the association between the PHDS and weight or BMI adjusted additionally with the mediator (each metabolite at a time).
[3]Mediation effect indicating the mediating role of the mediator.
[4]Proportion mediated indicating the proportion that mediator explained the association between the PHDS and weight or BMI.

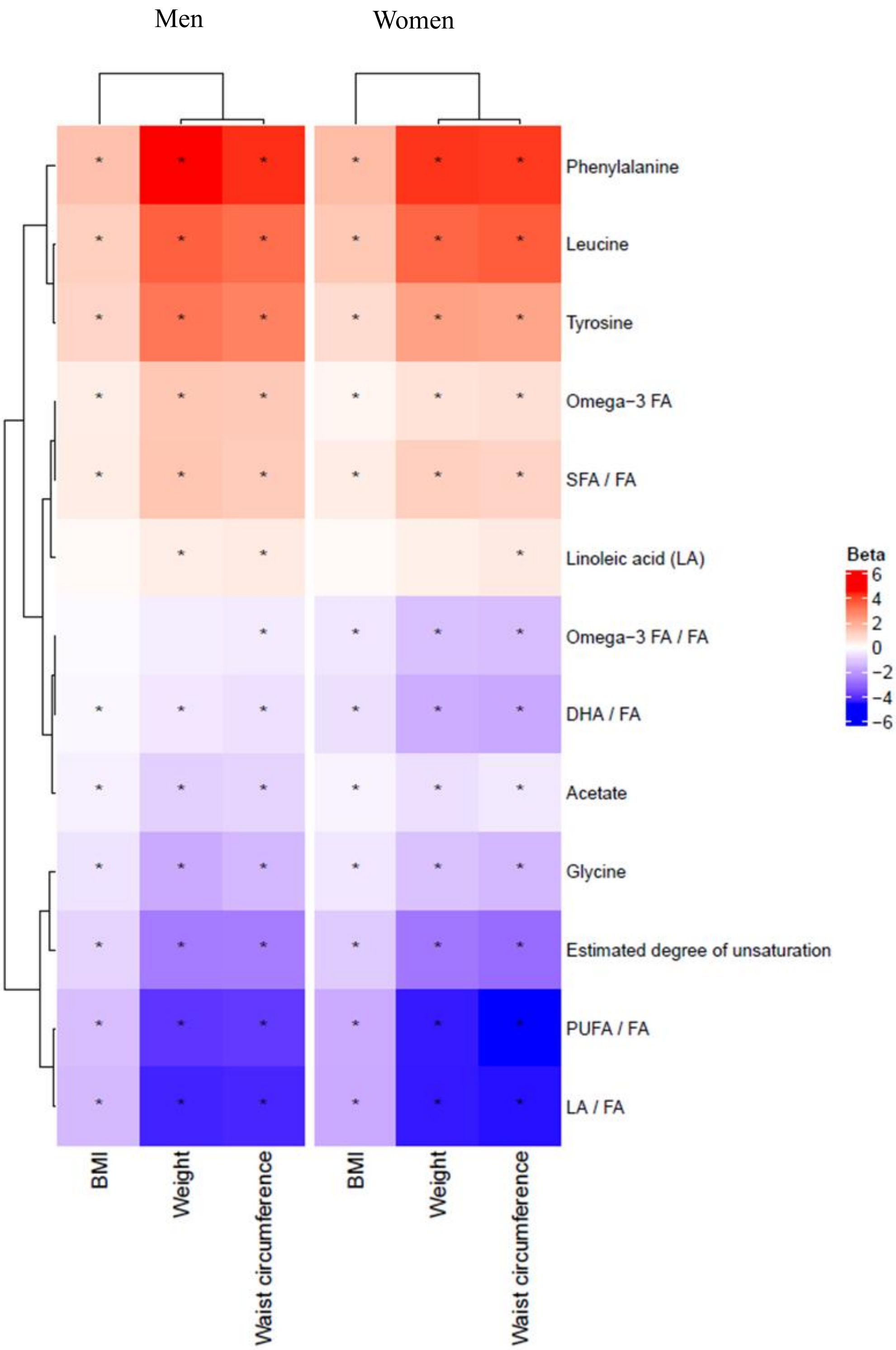

Supplemental figure 2. Adjusted (age, cohort, leisure-time physical activity, smoking, and use of cholesterol-lowering medication) linear associations (beta coefficients) between selected metabolites (found statistically significantly associated with the PHDS) and anthropometric measures (body mass index (BMI), weight, and waist circumference) in men and women in the pooled main dataset of FinHealth 2017 and DILGOM 2007 studies. Statistically significant false discovery rate (FDR) corrected p-values (significance level of 0.05) indicated with asterisk (*). DHA, docosahexaenoic acid; FA, fatty acid; LA, linoleic acid; PUFA, polyunsaturated fatty acid; SFA, saturated fatty acid.

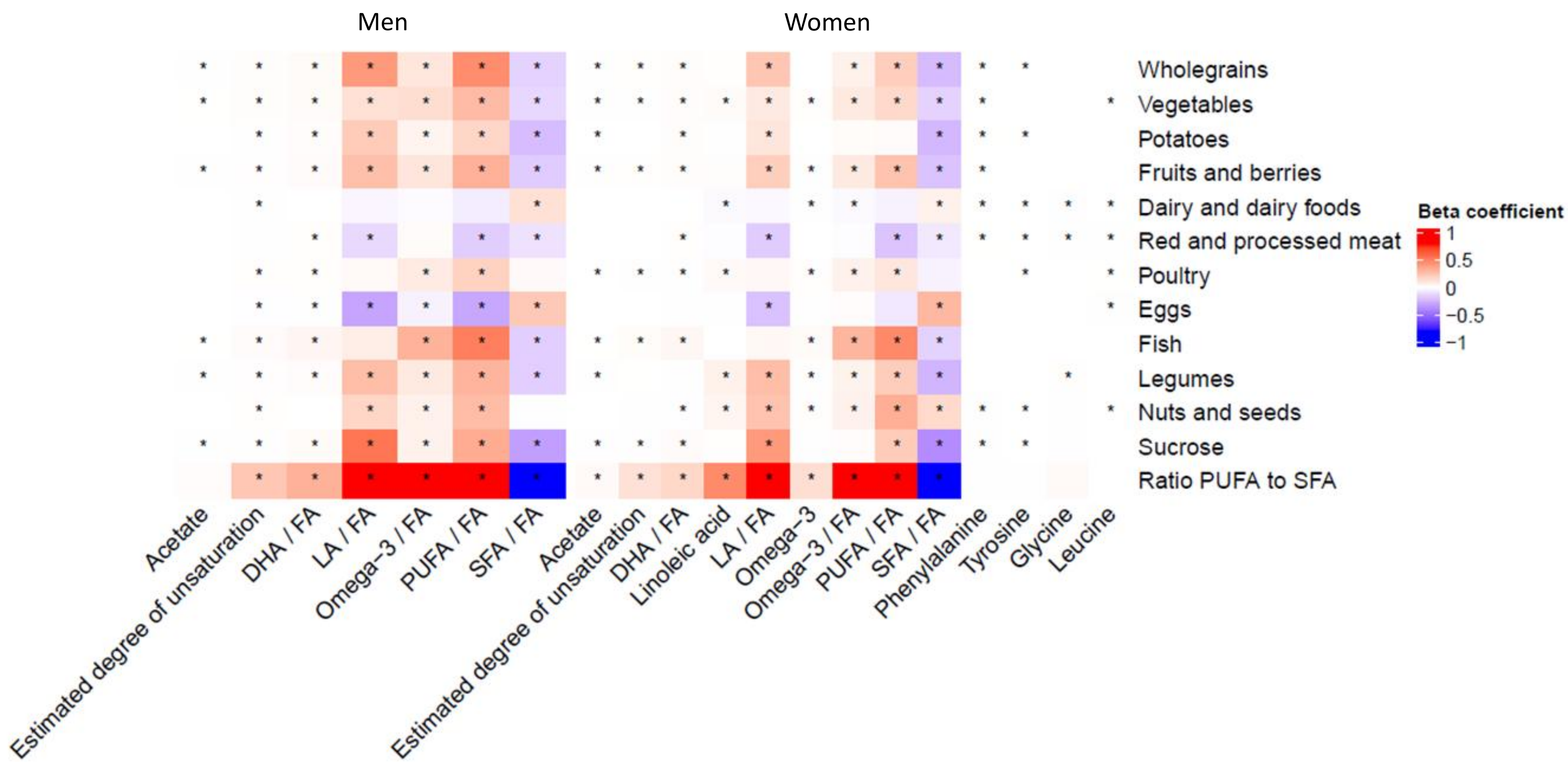

Supplemental figure 3. Adjusted (age, cohort, leisure-time physical activity, smoking, and use of cholesterol-lowering medication) linear associations (beta coefficients) between selected metabolites (found statistically significantly associated with the PHDS) and each component of the PHDS in men and women in the pooled main dataset of FinHealth 2017 and DILGOM 2007 studies. Statistically significant false discovery rate (FDR) corrected p-values (significance level of 0.05) indicated with asterisk (*). DHA, docosahexaenoic acid; FA, fatty acid; LA, linoleic acid; PUFA, polyunsaturated fatty acid; SFA, saturated fatty acid

Supplemental table 2. Baseline and follow-up characteristics of all participants and according to the studies included in the replication dataset of FINRISK 2012 and Health 2000 and 2011 studies.

	All		FINRISK 2012		Health 2000	
	Men	Women	Men	Women	Men	Women
**Baseline**						
Number of participants	4674 (46)	5568 (54)	2126 (46)	2477 (54)	2548 (45)	3091 (55)
Age, years	52 (14)	52 (15)	53 (14)	52 (14)	51 (14)	53 (15)
Leisure-time physical activity[1]						
Inactive, %	23	23	19	20	26	26
Moderate, %	51	55	49	51	52	59
Active, %	27	22	32	29	22	15
Smoking status						
Current smoker, %	26	19	20	15	32	21
Former smoker, %	32	16	33	20	31	14
Non-smoker, %	42	65	47	65	37	65
Cholesterol medication						
Users, %	13	9	20	14	7	6
Dietary information						
Daily energy intake, KJ	10250 (3544)	8873 (3140)	10484 (3607)	8692 (3019)	10055 (3480)	9199 (3216)
Planetary Health Diet Score[2]	3.3 (1.2)	3.7 (1.3)	3.3 (1.2)	3.9 (1.4)	3.3 (1.1)	3.6 (1.2)
Anthropometric information						
Height, cm	176 (7)	163 (7)	177 (7)	163 (6)	176 (7)	162 (7)
Weight, kg	85 (14)	71 (14)	86 (14)	71 (14)	84 (14)	70 (14)
BMI, kg/m^2	27 (4)	27 (5)	27 (4)	27 (5)	27 (4)	27 (5)
WC, cm	98 (12)	88 (13)	98 (12)	87 (14)	98 (11)	88 (13)
**Follow-up**					Health 2011	
Weight change, %[3]					1.3 (8.4)	2.8 (10.3)
BMI change, %[4]					1.8 (8.3)	3.6 (10.3)
WC change, %[5]					2.4 (7.3)	3.4 (9.2)

KJ, kilojoule; BMI, body mass index; WC, waist circumference

Data is presented as means with standard deviations (SD) for continuous variables or percentages for categorical variables.

[1]Self-reported leisure-time physical activity: inactive (light activities such as reading), moderately active (walking/cycling at least 4 h/wk), active (physically demanding activities at least 3 h/wk)

[2]Score could range from 0 to 13 points.

[3]n_{men} = 1959, n_{women} = 2417

[4]n_{men} = 1958, n_{women} = 2416

[5]n_{men} = 1678, n_{women} = 2037

Supplemental table 3. Baseline consumption of the Planetary Health Diet Score components in the pooled replication dataset of FINRISK 2012 and Health 2000 studies.

	Mean consumption (SD)		Scoring the PHDS
	Men	Women	
**Baseline**			
Planetary Health Diet Score	3.3 (1.2)	3.7 (1.3)	Maximum 13 p
Wholegrains[1], g/d	74 (47)	70 (43)	1 p ≥ 232 g/d<br>0 p < 232 g/d
Vegetables, g/d	232 (178)	294 (204)	1 p ≥ 300 g/d<br>0 p < 300 g/d
Potatoes, g/d	168 (121)	139 (111)	1 p ≤ 100 g/d<br>0 p > 100 g/d
Fruit and berries, g/d	167 (169)	240 (208)	1 p ≥ 200 g/d<br>0 p < 200 g/d
Dairy and dairy foods[2], g/d	936 (504)	894 (448)	1 p ≤ 500 g/d<br>0 p > 500 g/d
Red and processed meat, g/d	162 (93)	116 (75)	1 p ≤ 28 g/d<br>0 p > 28 g/d
Poultry, g/d	30 (37)	36 (44)	1 p ≤ 58 g/d<br>0 p > 58 g/d
Eggs, g/d	33 (30)	28 (28)	1 p ≤ 25 g/d<br>0 p > 25 g/d
Fish, g/d	54 (45)	49 (46)	1 p ≤ 100 g/d<br>0 p > 100 g/d
Legumes, g/d	15 (17)	15 (23)	1 p ≥ 75 g/d<br>0 p < 75 g/d
Nuts and seeds, g/d	2 (6)	4 (9)	1 p ≥ 25 g/d<br>0 p < 25 g/d
Sucrose, g/d	52 (28)	49 (28)	1 p ≤ 31 g/d<br>0 p > 31 g/d
Ratio PUFA to SFA	1.3 (0.3)	1.3 (0.3)	1 p ≥ 2.0<br>0 p < 2.0

PHDS, Planetary Health Diet score; PUFA, polyunsaturated fatty acids; SFA, saturated fatty acids
[1]Rye, oats, and barley were chosen to reflect whole grains in the Finnish diet.
[2]Dairy and dairy products included liquid milk and other dairy products (e.g., cheese and cream) as milk equivalents: for cream and ice cream equivalent was 2.7, for cheese products 5, and for butter 6.5 according to the equivalents used in the Stockholm Resilience Centre Nordic report [7].

Supplemental table 4. Linear association between the Planetary Health Diet Score (PHDS) and anthropometrics by sex in the pooled replication dataset of FINRISK 2012 and Health 2000 and 2011 studies.

		Men					Women				
		95% CI					95% CI				
		ß	lower	upper	p[1]	FDR[2]	ß	lower	upper	p	FDR
Weight, kg	Model 1	0.16	-0.15	0.46	0.32	0.43	-0.41	-0.67	-0.15	0.002	**0.01**
	Model 2	0.13	-0.17	0.43	0.40	0.46	-0.28	-0.54	-0.02	0.04	0.10
BMI, kg/m^2	Model 1	0.05	-0.05	0.15	0.35	0.47	-0.17	-0.27	-0.07	< 0.001	**< 0.001**
	Model 2	0.04	-0.06	0.14	0.43	0.49	-0.12	-0.22	-0.02	0.02	0.053
WC, cm	Model 1	0.0005	-0.28	0.28	1.00	1.00	-0.56	-0.82	-0.30	< 0.001	**< 0.001**
	Model 2	0.02	-0.25	0.29	0.89	1.00	-0.40	-0.65	-0.15	0.002	**0.01**
Weight change, %	Model 1	-0.02	-0.35	0.31	0.91	1.00	-0.06	-0.39	0.27	0.72	1.00
	Model 2	-0.0002	-0.33	0.33	1.00	1.00	-0.03	-0.36	0.30	0.85	1.00
BMI change, %	Model 1	-0.03	-0.36	0.30	0.85	0.93	-0.08	-0.41	0.26	0.66	0.93
	Model 2	-0.02	-0.35	0.31	0.91	0.93	-0.05	-0.38	0.28	0.77	0.93
WC change, %	Model 1	-0.17	-0.50	0.15	0.30	0.62	-0.12	-0.45	0.21	0.48	0.62
	Model 2	-0.13	-0.46	0.20	0.44	0.62	-0.08	-0.41	0.24	0.62	0.62

BMI, body mass index; FDR, false discovery rate; WC, waist circumference
[1]P-value for linear association.
[2]FDR by Benjamini-Hochberg procedure, FDR-value < 0.05 was considered statistically significant (significant results bolded).
Model 1 adjusted with age and cohort, and further anthropometric change with baseline measure (weight/BMI/WC) depending on which of these was examined. Weight change was additionally adjusted for baseline height.
Model 2 is Model 1 further adjusted for leisure-time physical activity, smoking, and use of cholesterol-lowering medication.

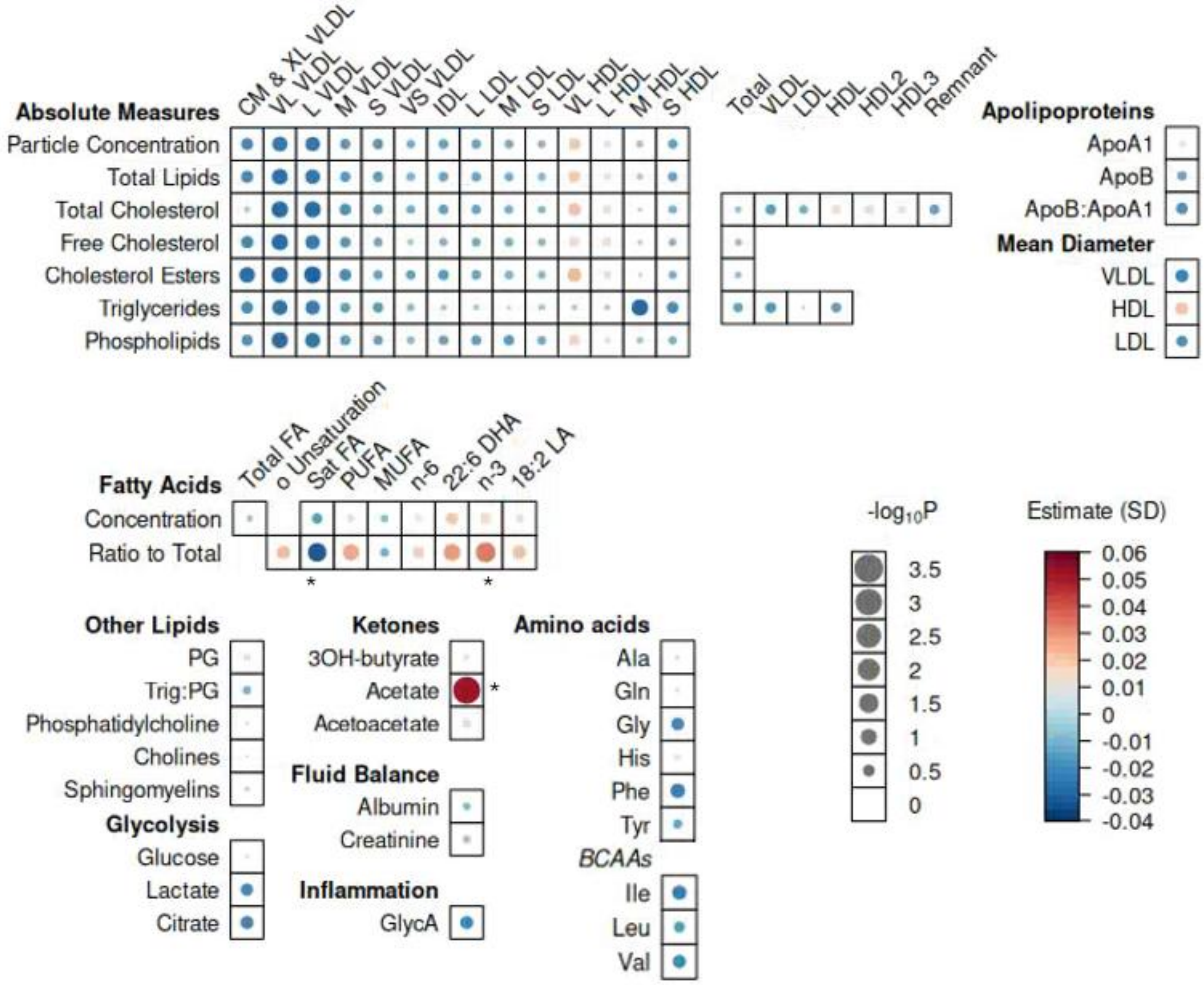

Supplemental figure 4. Linear associations between the Planetary Health Diet Score (PHDS) and 158 metabolites set to the z-score (mean 0, SD 1) for women in the pooled replication dataset of FINRISK 2012 and Health 2000 studies. The association adjusted with age, cohort, leisure-time physical activity, smoking, and use of cholesterol-lowering medication. Statistically significant false discovery rate (FDR) corrected p-values (< 0.05) indicated with asterisk (*). Ala, alanine; ApoA1, apolipoprotein A-I; ApoB, apolipoprotein B; BCAA, branched-chain amino acid; DHA, docosahexaenoic acid; FA, fatty acid; Gln, glutamine; Gly, glycine; GlycA, glycoprotein acetyls; HDL, high-density lipoprotein; His, histidine; IDL, intermediate-density lipoprotein; Ile, isoleucine; LA, linoleic acid; LDL, low-density lipoprotein; Leu, leucine; MUFA, monounsaturated fatty acid; Phe, Phenylalanine; PG, phosphoglyceride; PUFA, polyunsaturated fatty acid; SFA, saturated fatty acid; Trig, triglycerides; Tyr, tyrosine; Val, valine; VLDL, very-low-density lipoprotein.

Supplemental table 5. Mediating role of selected metabolites in the association between the Planetary Health Diet Score (PHDS) and baseline waist circumference (WC) for women in the pooled replication dataset of FINRISK 2012 and Health 2000 studies.

	Total effect[1] (WC ~ PHDS)		Direct effect[2] (WC ~ PHDS + metabolite)				
Metabolite	ß (SE)	FDR	ß (SE)	FDR	Mediation effect[3]	Proportion mediated, %[4]	FDR
Acetate			-0.38 (0.002)	0.01	-0.02	5.1	**0.020**
Omega-3/FA	-0.40 (0.002)	0.006	-0.37 (0.002)	0.01	-0.03	6.8	**0.003**
SFA/FA			-0.36 (0.002)	0.01	-0.04	8.2	**0.007**

FA, fatty acid; FDR, false discovery rate; SFA, saturated fatty acid.
Results are presented as beta coefficients with standard errors (SE), and FDR corrected p-values with significance level of 0.05 (significant results bolded).
All associations adjusted with age, cohort, leisure-time physical activity, smoking, and use of cholesterol-lowering medication.
[1]Total effect indicating the association between the PHDS and WC without the mediator (metabolite).
[2]Direct effect indicating the association between the PHDS and WC adjusted additionally with the mediator (each metabolite at a time).
[3]Mediation effect indicating the mediating role of the mediator.
[4]Proportion mediated indicating the proportion that mediator explained the association between the PHDS and WC.

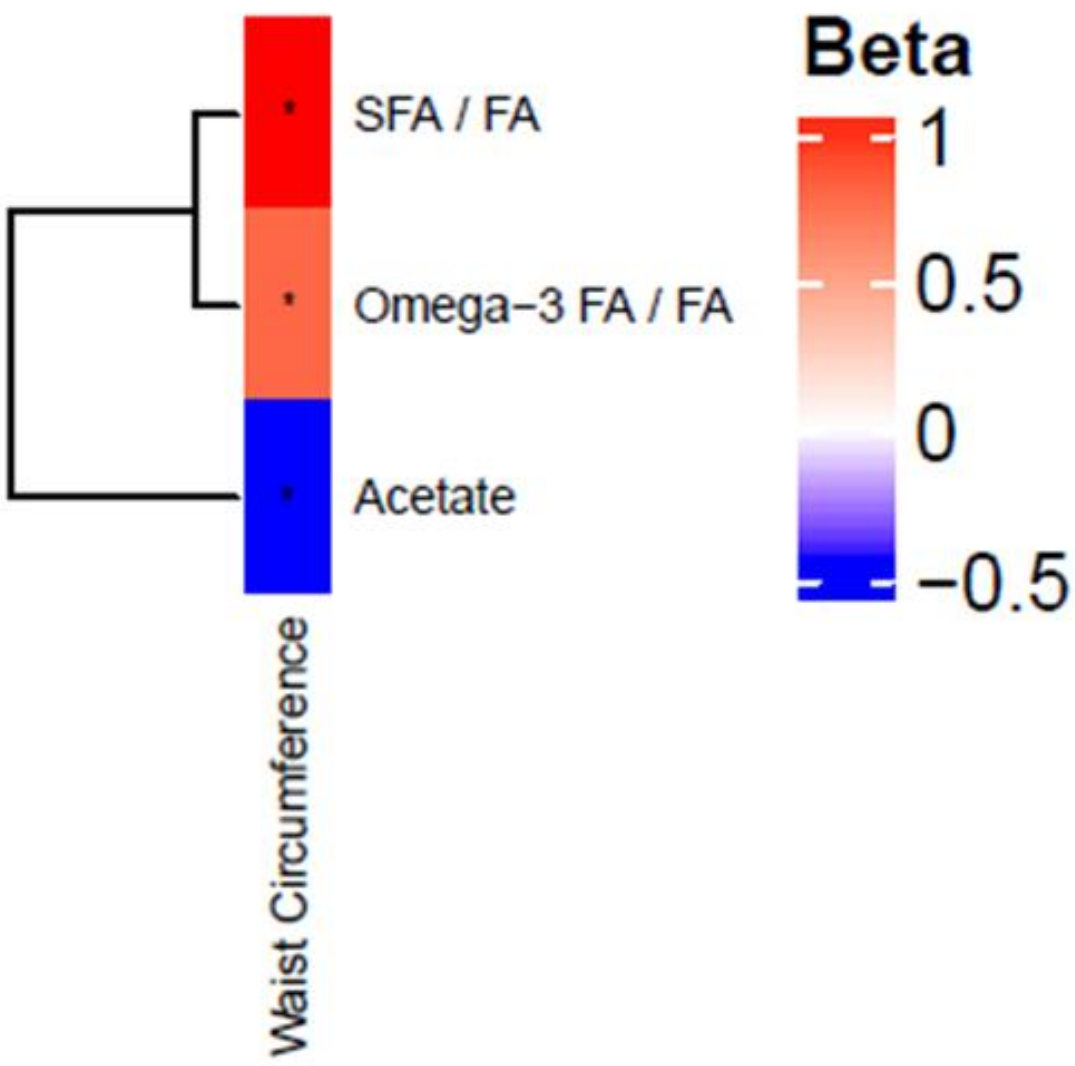

Supplemental figure 5. Adjusted (age, cohort, leisure-time physical activity, smoking, and use of cholesterol-lowering medication) linear associations (beta coefficients) between selected metabolites (found statistically significantly associated with the PHDS) and waist circumference in women in the pooled replication dataset of FINRISK 2012 and Health 2000 studies. Statistically significant false discovery rate (FDR) corrected p-values (significance level of 0.05) indicated with asterisk (*). FA, fatty acid; SFA, saturated fatty acid.

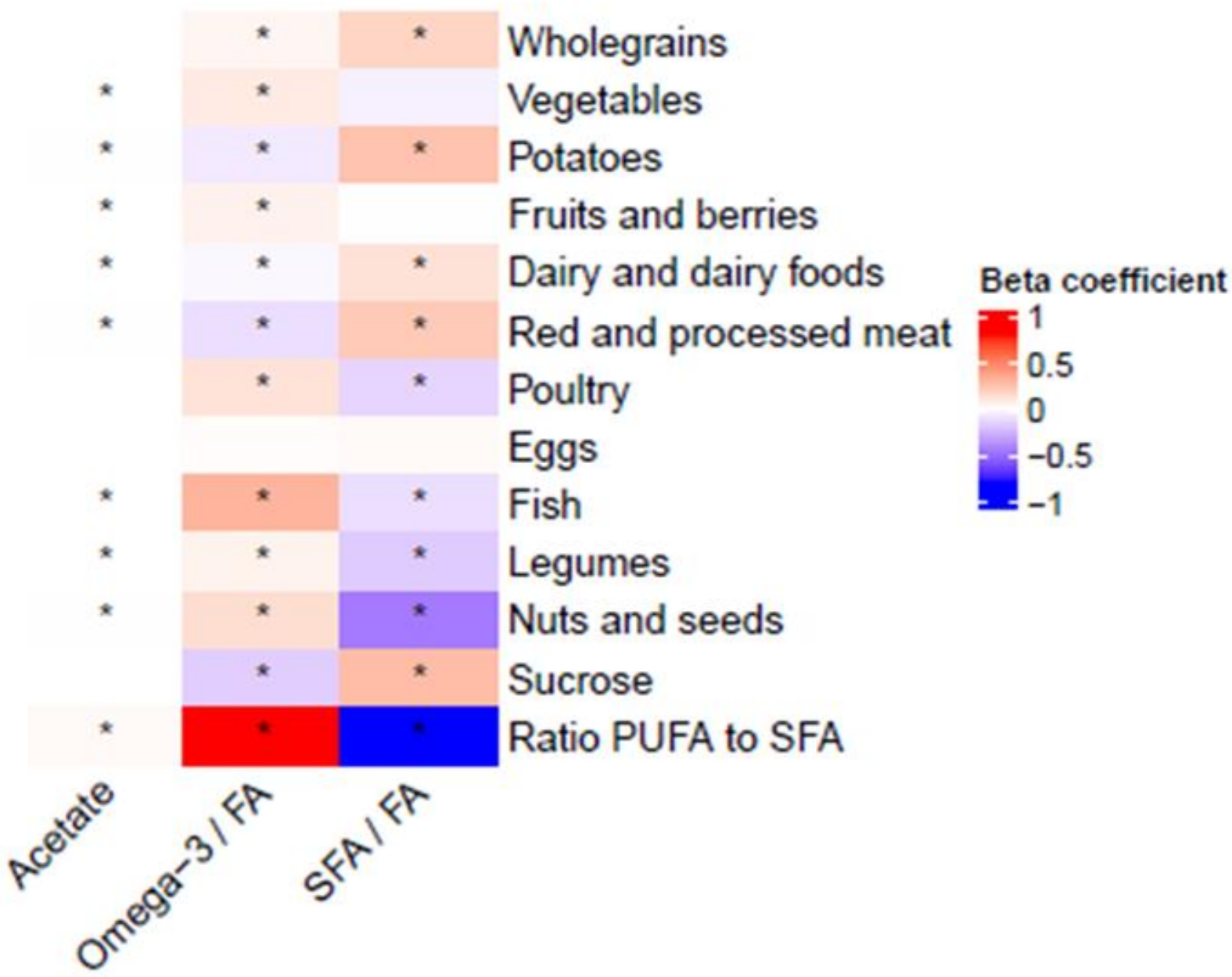

Supplemental figure 6. Adjusted (age, cohort, leisure-time physical activity, smoking, and use of cholesterol-lowering medication) linear associations (beta coefficients) between selected metabolites (found statistically significantly associated with the PHDS) and each component of the PHDS in women in the pooled replication dataset of FINRISK 2012 and Health 2000 studies. Statistically significant false discovery rate (FDR) corrected p-values (significance level of 0.05) indicated with asterisk (*).FA, fatty acid; SFA, saturated fatty aci

References

1. Heistaro S, ed. Methodology report. Health 2000 survey. Publications of the National Public Health Institute. Report B26/2008. Helsinki: National Public Health Institute. Available from: https://urn.fi/URN:NBN:fi-fe201204193320
2. Lundqvist A, Mäki-Opas T, eds. Health 2011 survey – methods. Finnish National Institute for Health and Welfare. Report 8/2016. Tampere. Available from: https://urn.fi/URN:ISBN:978-952-302-669-8
3. Konttinen, H, Männistö, S, Sarlio-Lähteenkorva, S, Silventoinen K, Haukkala A. Emotional eating, depressive symptoms and self-reported food consumption. A population-based study. Appetite 2010;54:473–9.
4. Kanerva N, Harald K, Männistö S, Kaartinen NE, Maukonen M, Haukkala A, et al. Adherence to the healthy Nordic diet is associated with weight change during 7 years of follow-up. Br J Nutr 2018; 120(1): 101–10.
5. Borodulin K, Saarikoski L, Lund L, Juolevi A, Grönholm M, et al. Kansallinen FINRISKI 2012 -terveystutkimus - Osa I: Tutkimuksen toteutus ja menetelmät (Abstract available in English). Report 22/2013. Finnish Institute for Health and Welfare. Availbale from: https://urn.fi/URN:ISBN:978-952-302-053-5.
6. Borodulin K, Sääksjärvi K. FinHealth 2017 Study: Methods. Finnish Institute for Health and Welfare. Report 17/2019. Helsinki, Finland. Available from: http://urn.fi/URN:ISBN:978-952-343-449-3
7. Wood A, et al. Erratum: Nordic food systems for improved health and sustainability – baseline assessment to inform transformation. Stockholm Resilience Centre, Stockholm University. 2019. Available from: https://www.stockholmresilience.org/download/18.8620dc61698d96b1901719c/1561013818461/Erratum_Nordic%20report_14-6-19.pdf.
